# The development of support intuitions and object causality in juvenile Eurasian jays (*Garrulus glandarius*)

**DOI:** 10.1038/srep40062

**Published:** 2017-01-05

**Authors:** Gabrielle Davidson, Rachael Miller, Elsa Loissel, Lucy G. Cheke, Nicola S. Clayton

**Affiliations:** 1 Department of Psychology, University of Cambridge, Cambridge, CB2 3EB, UK; 2 Biological, Earth and Environmental Sciences, University College Cork, Ireland

## Abstract

Knowledge about the causal relationship between objects has been studied extensively in human infants, and more recently in adult animals using differential looking time experiments. How knowledge about object support develops in non-human animals has yet to be explored. Here, we studied the ontogeny of support relations in Eurasian jays (*Garrulus glandarius*), a bird species known for its sophisticated cognitive abilities. Using an expectancy violation paradigm, we measured looking time responses to possible and impossible video and image stimuli. We also controlled for experience with different support types to determine whether the emergence of support intuitions is dependent upon specific interactions with objects, or if reasoning develops independently. At age 9 months, birds looked more at a tool moving a piece of cheese that was not in contact than one that was in direct contact. By the age of 6 months, birds that had not experienced string as a support to hold up objects looked more at impossible images with string hanging from below (unsupported), rather than above (supported). The development of support intuitions may be independent of direct experience with specific support, or knowledge gained from interactions with other objects may be generalised across contexts.

The ability to reason about objects and how they relate to one another is an important part of interacting with the physical world. By knowing that an object will fall if not supported from below, one can ensure the correct placement of objects on stable surfaces. Intuitions about support may be dependent on developing rules about observable support (e.g. other objects) and unobservable phenomena (e.g. gravity). Objects can interact in several different ways, and studies with human infants indicate that they develop intuitions about different categories of support at different ages^1^,^2^,^3^,^4^. At the age of 3 months, infants understand the basic concept that objects must be in contact in order to be supported. By 4.5 months of age, they recognise that the positioning of contact is also important (i.e. an object needs to be underneath another object, rather than in contact along the vertical edges)^2^. At 6.5 months of age, they distinguish between the amount of contact that is necessary for one object to be supported by another. Objects can also interact when colliding with one another. In ‘launching events’, objects can be shown to move relative to each other in possible and impossible ways. It is not until 6–7 months of age that infants are sensitive to no contact collision events (i.e. the second object moves before an approaching object makes physical contact with it) compared to contact collision events (i.e. the second object moves only once the first object collides with it)^5^,^6^.

Comparative studies on adult animals has shown that chimpanzees (*Pan troglodytes*)^7^,^8^ and monkeys (*Macaca fuscata*)^8^ distinguish between contact vs no contact and amount of contact, but do not distinguish between the position (i.e. type) of contact. These studies suggest that apes and monkeys differ from infants in their understanding of the support^7^. A phylogenetically distant species, the rook (*Corvus frugilegus*) has also been tested using a similar paradigm, and perceived these three categories of support in the same way as 6.5 month old infants^9^.

There is growing evidence that corvids (e.g. crows, rooks and jays) have sophisticated levels of causal reasoning in the physical domain (i.e. the understanding of how and why cause and effect relationships work), e.g. refs 10, 11, 12, 13. New Caledonian crows not only use tools to gain access to grubs in tree crevices in the wild^14^, they can choose the correct tool shape^15^,^16^ or manipulate current tools to reflect the current task^16^,^17^,^18^. Moreover, rooks and Eurasian jays (*Garrulus glandarius*) are species that do not typically use tools in the wild, yet they are capable of doing so when given the opportunity in the lab^11^,^13^,^19^. In the Aesop’s’ fable task, a tube of water contains a floating worm that is out of reach. In order to raise the water level, rooks prefer to drop large (more efficient) stones instead of small stones^11^, and Eurasian jays drop sinking (functional) objects, rather than floating (non-functional) objects^13^, suggesting they have a causal understanding of objects and their physical properties. One interpretation of these results is that corvids may reason about object support and that recognising different types of object relations is advantageous to tool-using species. However, given that rooks and Eurasian jays are non-tool users outside of the lab, it has been suggested that corvids share a general intelligence, which is independent of their experience and current ecological niches^20^,^21^.

Cognitive reasoning in relation to the physical world may develop through perceptual and motor experiences, though it has also been proposed that cognition first emerges independently from these experiences, and that infants have intuitive theories that form their “core knowledge” about the world. This knowledge becomes more sophisticated over time in an incremental fashion^3^,^4^,^22^. Evidence for core knowledge is further supported by studies with chicks (*Gallus gallus*), where early rearing experience can be controlled for in this precocial bird. Newly hatched chicks have intuitions about the physical properties of imprinted objects that can or cannot be occluded behind screens^23^, can reorient based on physical features of geometric structures they have not previously experienced^24^, and show a concept for numerosity when associating with a choice of differing numbers of objects (perceived as social agents through imprinting)^25^ (reviewed in ref. 26). There is also evidence to suggest that experience may contribute to how human infants respond to support relations. When 4.5 month old babies are shown how impossible support is maintained by a hidden experimenter’s hand, they no longer recognise this as unusual^2^. The contribution of experience and core cognitive capacities may not be mutually exclusive. The emergence of tool use in juvenile New Caledonian crows appears to have a genetic component, while the efficacy and preference of tool shapes is also influenced through social learning from a demonstrator^27^,^28^. How corvids acquire knowledge about support relations, whether they share similar developmental milestones as human infants, and how experience interacts with development remains unknown.

Determining how young infants and animals perceive object relations has been studied by measuring subjects’ looking time in response to stimuli, and has been widely used across species including infants^1^, birds^7^, primates^9^, and horses^29^. This expectancy violation measures subjects’ interest and assumes they will look more when presented with stimuli that are surprising. Therefore, by this rationale, if individuals look longer at an impossible support event, we can infer that this violates their current understanding of causal relationships between objects. However, this expectancy violation paradigm is not without criticisms, as some authors have suggested that increased looking times may be in response to the novelty of the perceptual features, rather than any underlying cognitive basis for support intuitions (e.g. ref. 7). Therefore impossible events may be perceived as novel and salient, rather than perceived as something unusual in the context of expected object relations. Nevertheless, this paradigm is a useful tool for investigating how animals respond to visual stimuli, and is well suited to explore the development of their understanding of the physical world. Corvids in particular are ideal subjects to explore these questions given their cognitive and tool use abilities (e.g. refs 10,11,13), and relatively short developmental period.

In this study, we investigated how juvenile Eurasian jays reason about support and how this knowledge develops from age 3–9 months of age using an expectancy violation paradigm. We approached this question using two sets of possible and impossible stimuli –videos in which a piece of cheese moved relative to a rake-like tool, and static images in which a piece of cheese was supported (or not supported) by a box. More specifically, the videos investigated the birds’ looking responses to contact vs no contact and type of contact (incorrectly behind the direction of the rake movement) (Fig 1). The static images investigated the birds’ looking responses to three categories of contact in order to establish any core knowledge of support at an early age: contact vs no contact, amount of contact, and type of contact (incorrectly along vertical edges). The static images were presented again at 6 months and were controlled for visual novelty by duplicating each support category: one image with a supporting string above the cheese, and one image with a non-supporting string below the cheese (Fig. 2). This design controlled for responses being due to the novelty of the image, because each pairwise cheese condition appeared identical aside from the position of the string, and therefore the support interaction.

To determine whether or not experience of visual and/or hidden support was necessary for the development of support intuitions, we split the birds into two experience groups. In one group (the “string” group), the birds interacted with fruit that was suspended by a string from their perch (visible support), and in the second group (the “nail” group), the birds interacted with fruit that was suspended on a vertical wooden board by a nail (hidden support). If birds recognised impossible causal interactions between objects, we expected birds to look longer at the no contact, insufficient contact and incorrect contact conditions. If the acquisition of support intuitions is determined by fixed development periods, we expected both nail and string groups to respond in the same way to the impossible conditions at the same developmental stages. If experience of visible and hidden support influenced responses to support stimuli, we expected the nail group to look longer at the no contact and insufficient contact conditions regardless of string position, whereas the string group would look longer at all impossible contact categories only in the string down positions. Furthermore, the nail group should not find the vertical contact surprising, regardless of string position, owing to their experience of hidden support.

One challenge in using visual presentations and looking times with birds is that their visual systems differ from humans and primates (for review see ref. 30). Determining where a bird is looking is not as straightforward as it is with animals that have forward facing eyes because the field of vision in birds is much wider than primates and comprises of both monocular and binocular vision^31^. To accurately measure where birds are looking, we adopted the peep hole method whereby birds can observe stimuli located behind a barrier by positioning their eye in front of a small hole^9^,^32^,^33^.

An additional challenge comes from the possibility that what we see on a screen may not be perceived by birds in the same way. Flicker fusion rate (the threshold at which a blinking light is seen as continuous) is relatively higher in birds such as chickens (*Gallus gallus*)^34^,^35^ than humans^36^. To account for this, our images and videos were displayed at a refresh rate of 120 hz. Videos were recorded and played at 120 frames per second, a presentation frequency that is likely over the minimum required for birds to perceive motion as smooth^37^. Moreover, to test whether birds were attending to the screen and would perceive objects in a similar manner to when observed in real life, we trained the birds on an associative learning task using live objects and tested whether this association could be transferred to objects displayed on a monitor.

## Results

### Associative learning task

12 out of the13 birds tested selected the correct (i.e. previously rewarded) object four times in a row in the live object choice, which was significantly more often than would be expected by chance (p = 0.0034). Of the 12 birds to receive a choice of objects displayed on the monitor, 10 birds selected the correct object, which was significantly more often than would be expected by chance (p = 0.039).

### Subject participation

Subject participation varied across experimental sessions. Some birds did not participate in some or all of the stimuli treatments (see Supplementary Information for full breakdown of subject participation). For the video experiment, 15 subjects participated at age 3 months, 13 at age 4 months, 14 at age 6 months and 10 at age 9 months (mean number of birds per video = 13.7, 9.5, 11.7, 7.5, respectively). For the image experiment, 15 subjects participated at age 3 and age 6 months (7 string group, 8 nail group) (mean number of birds per image = 14.8, 6.6, 7.1, respectively).

### Video presentations

There was a main effect in the videos with the cheese outside the rake where birds looked significantly less at the no contact videos compared to the control (Est. −0.53 ± 0.27, z = −2.44, p = −0.015). A significant interaction between age and support type showed that by age 9 months, birds looked significantly longer at the no contact video (Est. 1.03 ± 0.36, z = 2.84, p = 0.005), and a non-significant trend indicated that birds looked less at incorrect contact at age 4 months (Est. −0.58 ± 0.33 S.E., z = −1.75, p = 0.08). There was no effect of support when the cheese was inside the rake, regardless of age (incorrect contact, Est. 0.04 ± 0.11, z = 0.35, p = 0.73; no contact, Est. – 0.02 ± 0.11, z = −0.22, p = 0.82).

**Figure 3:**
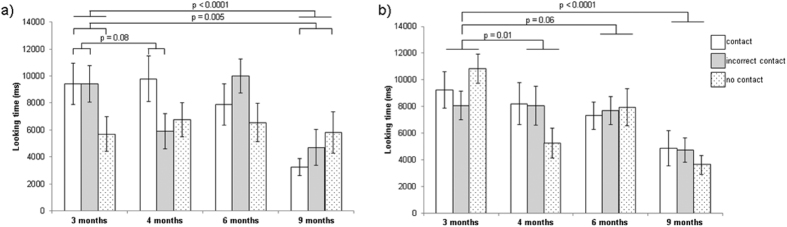
Looking time results for videos stimuli across all age stages (3, 4, 6 and 9 months). Cheese was positioned a) outside of the rake, GLMM age*no support Est. 1.03 ± 0.36, z = 2.84, p = 0.005; age*incorrect support; age 9 months Est. −1.00 ± 0.25, z = −3.64, p = 0.0003 and b) cheese was positioned inside the rake. GLMM age 4 months Est. −0.33 ± 0.13 SE, z = −2.57, p = 0.01; 6 months Est. −0.23 ± 0.12 SE, z = −1.94, p = 0.05; 9 months Est. −0.93 ± 0.03 SE, z = −6.44, p < 0.0001. Contact and age 3 months were set as the reference categories. Whiskers denote 1 standard error.

There was a significant effect of presentation order whereby birds looked less across presentations (cheese outside Est. 0.14 ± 0.03, z = −4.69, p < 0.0001; cheese inside Est. −0.10 ± 0.03, z = −3.71, p = 0.0002). Overall, birds decreased their looking time as they grew older. At age 9 months, birds looked significantly less at the videos than at 3 months when cheese was outside the rake (Est. −1.00 ± 0.25, z = −3.64, p = 0.0003). Birds looked significantly less than at 3 months when the cheese was inside the rake at 4 months (Est. −0.33 ± 0.13, z = −2.57, p = 0.01) and at 9 months (Est. −0.93 ± 0.03, z = −6.44, p < 0.0001). They also tended to look less at 6 months (Est. −0.23 ± 0.12, z = −1.94, p = 0.05) (Fig. 3).

### Image presentations

**Figure 4:**
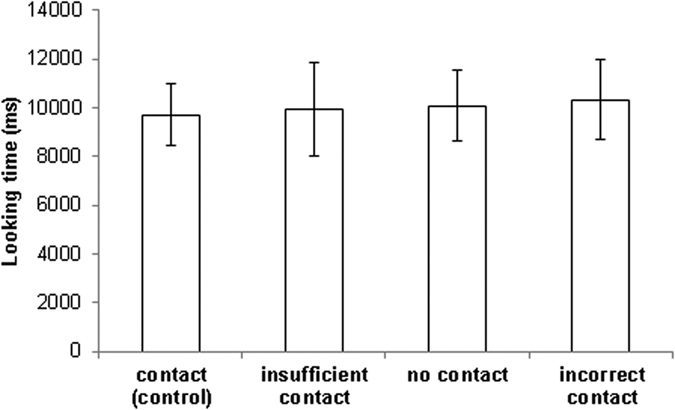
Looking times at age 3 months. GLMM insufficient support Est. 0.12 ± 0.2, z = 0.61, p = 0.54; no support Est. −0.11 ± 0.2, z = −0.57, p = 0.57; incorrect support Est. −0.29 ± 0.06, z = 0.22, p = 0.83). Contact (control) was set as the reference category. Whiskers denote 1 standard error.

At 3 months of age, looking times for the experimental images did not differ from the control image (insufficient support Est. 0.12 ± 0.2, z = 0.61, p = 0.54; no support Est. −0.11 ± 0.2, z = −0.57, p = 0.57; incorrect support Est. −0.29 ± 0.06, z = 0.22, p = 0.83). There was a significant effect of presentation order where birds looked less across successive trials (z = −4.88, p = < 0.0001) (Fig. 4).

**Figure 5:**
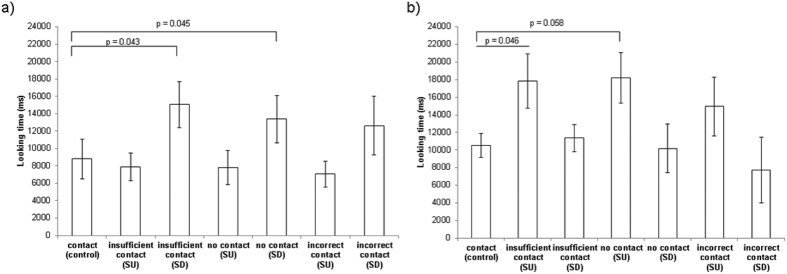
Looking times at age 6 months with string up (SU) and string down (SD) images. (**a**) nail group: GLMM no support SD (Est. 0.56 ± 0.28, z = 2.00, p = 0.045) and insufficient support SD (Est. 0.53 ± 0.26, z = 2.03, p = 0.043); (**b**) string group: GLMM no contact SU (Est. 0.56 ± 0.28, z = 1.99, p = 0.046) and insufficient support SU (Est. 0.53 ± 0.28, z = 1.89, p = 0.058). Contact (control) was set as the reference category. Whiskers denote 1 standard error.

At 6 months of age, the nail group looked significantly longer at no support string down, i.e. no support from box or string (Est. 0.56 ± 0.28, z = 2.00, p = 0.045) and at insufficient support string down (Est. 0.53 ± 0.26, z = 2.03, p = 0.043), but did not look longer at any of the other conditions relative to the control. By contrast, the string group looked significantly more at no support string up, i.e. cheese could be supported by string though not the box (Est. 0.56 ± 0.28, z = 1.99, p = 0.046) and tended to look longer at insufficient support string up (Est. 0.53 ± 0.28, z = 1.89, p = 0.058). They did not look longer at any other conditions. There was no effect of presentation order for either group (Fig. 5) (see Supplementary Information for full table of results).

## Discussion

Our results show that by 9 months of age, young Eurasian jays demonstrated some concept of the necessity of support for one object to move another, looking longer when the cheese was moved by a rake that was not in contact, but only when the cheese was positioned outside the rake. At 6 months old, the birds without experience of string-support demonstrated surprise to impossible, but not possible string support situations: the nail group looked longer at an image of cheese with no support and insufficient support when the string was hung down (i.e. neither the box nor string provided any support). These results are the first indications of how support intuitions develop in non-human animals, and show similarities to the looking time responses reported in adult chimpanzees and monkeys^7^,^8^, and in the case of no contact, 3 month old children^1^,^3^,^4^.

Basic concepts of support in human babies emerge early in life and show a consistent developmental trajectory across studies^3^,^4^, supporting the theory that core knowledge of object relations emerges before their concepts are refined as they mature^22^. Although the emergence of developmental milestones in cognitive abilities differ across species, the relative sequence of their emergence within some cognitive tasks such as object permanence, and gaze following, appears to be conserved (for reviews see refs 38,39). Using static images of supported and unsupported food items, we found no evidence of any core knowledge of support relations at age 3 months, but that longer looking times for both no contact and insufficient contact images had emerged by 6 months. This is in contrast to the developmental sequence found in infants, who acquire knowledge about the type of support before the amount of support.

In the context of horizontal movement, Eurasian jays’ support concepts appeared more in line with those of human infants^1^,^3^, as birds looked longer at interactions involving no contact than those involving contact, but showed no sensitivity to inadequate support by the age of 9 months. Moreover like human infants^5^,^6^, it is later in development that Eurasian jays show a sensitivity to contact vs no contact in the context of objects colliding with one another as opposed to objects supporting one another.

In our study, the frequency of repeat presentations was restricted because we found a significant drop in the duration of looks and a decrease in the number of birds motivated to participate as they matured. Therefore further study is required to confirm whether or not Eurasian jays acquire knowledge about support in an incremental fashion, and whether or not adult jays can reason about the type of contact (i.e. that objects can be supported from below, rather than the side). The latter was found to be the case in adult rooks, a closely related species that shares other cognitive abilities in the physical domain^11^.

Our study sought to determine whether support intuitions follow fixed developmental trajectories in Eurasian jays, and whether manipulating early experiences with specific object relationships would interact with these developmental stages. Developmental studies in corvids have investigated the sequence of when behaviours and underlying cognitive abilities emerge. In the absence of any experience with tools, juvenile New Caledonian crows show that motor actions are genetically inherited, while experience of observing demonstrators influences the types of tools they use^27^,^28^. Juvenile scrub-jays develop stage four object permanence (i.e. mental representations of hidden objects) before the emergence of tentative caching (hiding an object and immediately retrieving it)^40^. In the present study, the finding that birds without experience of string support (the nail group) looked longer at ‘impossible’ string support suggests that the development of support intuitions may be independent of experience, and perhaps due to fixed developmental milestones.

Although we controlled for subject experience by removing all objects hanging by strings in the aviary prior to the birds’ fledging the nest, the acquisition of support knowledge may have been triggered by (but not guided by) manipulation and observation of other objects in their aviary (sensu Spelke ref. 22). Perhaps more specific experiences with feathers falling during moulting, or leaves hanging from trees provided a knowledge base that could be generalised to other contexts. The finding that the nail group did not look more at the incorrect support image (i.e. the cheese made contact but only on a vertical edge) may be attributed to their prior experience with hidden nail support. Failure to look longer at impossible events may be due to subjects making inferences about alternative support^7^,^41^. However, it is difficult to disentangle this explanation from the possibility that juvenile birds just do not distinguish between different types of support, particularly because the string group (which did not have experience with hidden nail support) did not look more at incorrect support.

The expectancy violation paradigm assumes that looking time is an indication of surprise in response to stimuli that is contrary to one’s current understanding of the world, or to learn about novel stimuli. In this study, we attempted to control for novelty by duplicating each support condition and introduced two string positions that differed only in their location relative to the cheese. By doing so, we hoped to control for the salience of each pair-wise support image so that the birds’ looking responses would be more representative of support intuitions as opposed to responses to new visual features. Perhaps the most unexpected finding was that the string group looked longer in the string up conditions (i.e. where the string, but not the box, could potentially have provided support). We suggest that the string group was likely confounded by reward history during habituation to the string. To overcome the string group’s neophobia, worm rewards were placed on the fruit hung by a string. These birds may have formed an association between food reward and the orientation of string relative to the fruit, causing them to look longer (perhaps in expectation of a reward). This is particularly likely given their previous experience with orienting towards a stimuli to receive a reward in the associative learning task.

The string group did not look significantly more during all string up conditions; therefore string up alone was not a sufficient stimuli to elicit longer looking time responses. One possibility is that there was an additive effect of looking time with regards to the string orientation association and the birds’ surprise at specific support conditions (namely when the cheese was not in contact and had insufficient contact with the box). A more plausible interpretation is that the association with previous reward was specific to the exact visual features of the string holding an object with no support. While reward history remains a likely explanation for the looking time behaviour of the string group, we do not believe the results from the nail group were confounded by reward history, as this would predict those birds to have looked more at the incorrect support conditions. Our study demonstrates the importance of considering previous experience with stimuli and to use caution when interpret looking time as a measure of surprise. This is particularly relevant given the number of behaviour and comparative cognition studies adopting this paradigm (e.g. refs 9,29,42).

We demonstrated that birds could perceive images on the monitor similarly to live images in an associative learning task, and made every effort for the videos to appear natural. However, we cannot be certain how the videos were perceived by the birds and whether this could have inhibited their perception of the different categories of support. Moreover, we found that the positioning of the cheese relative to the rake influenced whether the birds looked more in the no contact condition, perhaps because the cheese was more distinct and salient when positioned on the outside, rather than the inside of the rake tool, or that the spatiotemporal features were more interesting to look at^43^. Furthermore, it is unknown what juvenile Eurasian jays expect regarding causal agency (but see ref. 44 for data on adult New Caledonian crows and^45^ on newly hatched chickens), as infants expect people, not objects to cause motion^46^,^47^. Therefore the rake tool moving on its own may have been perceived as an unexpected event in itself.

2.5 month old infants make errors when observing possible and impossible object relations, either by failing to distinguish between the two, or attending more to the possible events^4^,^48^. Baillargeon^4^ attributes responses to non-violation stimuli as an indication that infants are acquiring information about general physical rules. We found that from 3 months of age, birds looked less at the no contact, and at 4 months of age, tended to look less at the incorrect contact compared to full contact videos when the cheese was positioned outside the rake. We can only speculate as to why this may be case, but like human infants, this may be further support for their lack of physical knowledge at this age.

The growing body of work on animal cognition illustrates that corvids share many cognitive abilities with humans and other primates^20^,^49^. Here, we show that the ontogeny of support intuitions in Eurasian jays shares some parallels as well as some contrasts with human development. Comparative developmental studies which identify when cognitive capacities emerge, how they coincide with functional behaviours and if they are dependent on experiences with conspecifics and the environment are key to understanding the degree to which the ontogeny of cognition is shared across species.

## Methods

### Subjects

Eurasian jays participated in the study (though see results for samples sizes for each test condition). Birds were taken from the nest age 10–12 days post-hatching under a Natural England Licence 20140062 and hand-reared by an experienced bird breeder. At 4 weeks old, they were transported to the facility where they were housed in five 2 × 2 × 1 m indoor compartments, connected by open windows. At age 2 months, the birds were given access to the main outdoor aviary. During training and testing, birds were called into the indoor compartments and had the choice of participating in the task by entering the testing compartment. Birds were let out of the testing compartment into the outdoor aviary if they showed any signs of restlessness. Birds had access to water at all times and maintenance diet was provided, except one hour before testing to increase motivation to enter the testing compartments. At this point, they received wax worms and meal worms as a reward for moving between compartments, and access to the maintenance diet.

### Ethical Statement

All research has been approved under the European Research Council Executive Agency Ethics Team (application: 339993-CAUSCOG-ERR) and by the University of Cambridge Ethics Review Process, and has been conducted under the accordance of Home Office Regulations and the ASAB Guidelines for the Treatment of Animals in Behavioural Research and Teaching.

### Monitor set up and associative learning task

All stimuli were presented 1 metre from the peep hole on a BenQ 24” XL2430T LED monitor, connected to an Asus GL551JW-DS71-HID1 laptop with a GEFORCE GTX 960 M graphics card via a miniDisplay port. This set up was capable of transmitting 120 fps at 120 hz. Therefore we assumed that the videos would be perceived as a fluid motion without any flickering.

In an associative learning task, birds were trained to fly to a perch located in front of one of two objects – only one of which would be rewarded per subject. Once birds learned the association (see supplementary material for training phases and criterions), birds were presented with the same objects, but displayed on a monitor. If birds perceived the display similar to that of live objects, we expected the number of birds to choose correctly to be significantly greater than chance.

### Peep hole set up and training

At age 2 months, 16 juvenile Eurasian jays were trained to look into an adjacent compartment through a 1.5 cm diameter hole positioned 15 cm above a wooden perch. Birds were brought into the testing compartments individually and called to the perch by an experimenter located behind the peep hole. When a bird landed on a perch, a wax worm was held in front of the peep hole and the bird was rewarded for looking through the hole. Birds received a maximum of 6 worms per training session. This was repeated daily for two weeks.

Birds received one training session (as described above) the day prior to each experimental round, except at 9 months of age when they received 3 days of training. This was because the interval between previous training and/or peep hole experiments was longer than in previous rounds. Birds were not rewarded during experimental rounds, except at 6 months when they received one worm after they received all 6 video stimuli or all 7 image stimuli, and at 9 months after each daily session. This ensured the birds maintained motivation to return to the compartment for future experiments, as we found levels of restlessness increased throughout their development (pers. obs). Birds were not rewarded for looking at any particular image.

### Video and image stimuli

Videos of the rake moving the cheese were filmed at 120 fsp using a GoPro^®^ Hero4 Black. Hidden fishing wire was used to move the cheese relative to the rake without being in contact. Multiple takes were filmed and videos that matched in duration of rake movement (i.e. 6 seconds) were selected as the final stimuli. Videos were cut and edited in Blender™ 2.77a software to be 15 seconds total (the last 9 seconds of the rake and cheese being stationary).

Images were created by taking photographs of the cheese using a Sony nex 6 camera. The cheese and string were repositioned using Adobe^®^ Photoshop^®^ software. The cheese and string in all images were visually identical aside from their position relative to the box. Both videos and pictures were taken in the testing compartment to ensure lighting and shadows appeared natural. All birds were familiar with blocks of cheese, as this was provided as a food treat in the aviary on days prior to testing.

### Stimuli presentations

15 birds were individually brought into a testing compartment (2 m × 2 m × 1 m) that was visually isolated from the other birds, and from the monitor and the experimenter. The compartment contained three perches, one of which was located under the peep hole. In the adjacent compartment was the monitor, and between the laptop and experimenter was a cardboard divider. A black curtain covered the peep hole from the monitor side, and was raised by the experimenter when the stimuli were ready to present to the birds. The curtain was lowered at the end of the session. If a bird showed any signs of restlessness (i.e. flying between perches or making calls), they were released into the main aviary with the rest of the group and were brought in again either later in the day or more typically, the following day. Birds were tested for a maximum of 6 days.

A GoPro^®^ Hero4 Black was positioned adjacent to the peep hole to record looking time. The experimenter watched the bird’s behaviour through a live video feed using the GoPro^®^ mobile App. When the bird’s eye was level with the peep hole, the experimenter started the stimulus presentations. Static images were presented using Microsoft PowerPoint^®^ presentation graphics program, and videos were played using DivX^®^ video player.

**Figure 6:**
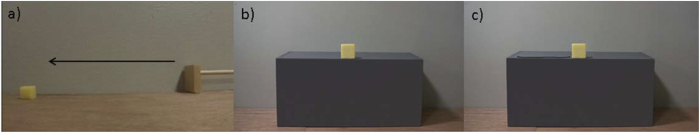
Familiarisation stimuli presented before each session. (**a**) videos, (**b**) core knowledge images, (**c**) string support images.

The birds received a familiarisation stimulus at the beginning of each session: the rake moved independently from the cheese for the videos (6 seconds), and the cheese (and string) positioned in the centre of the box for the images (5 seconds) (Fig. 6, supplementary materials). The purpose of the familiarisation stimuli was to control for birds responding to the first video/image as the most interesting (e.g. ref. 7). Birds received two test stimuli each session. Positional order was counterbalanced such that each stimulus was presented in each position an equal number of times. Videos were 15 seconds long followed by a black screen for 1 second. Images were displayed for 30 seconds followed by a black screen for 5 seconds. Each stimulus was played once, and the next stimulus was displayed following the black screen. If birds were not already looking through the peep hole between stimulus presentations, the experimenter paused the black screen and called them to the perch from behind the barrier, and restarted the sequence when the bird’s eye was level with the peep hole. Birds received videos at age 3, 4 6 and 9 months, and images at age 3 and 6 months of age (Table 1). Birds decreased participation and looking time across testing trials (see results), therefore to maintain motivation and reduce effects of habituation, we increased the intervals between videos after 4 months of age, and limited the presentation of static images to every 3 months. Due to a video recording failure in the image group, age 6 months, we repeated 2 image stimuli to 3 birds from nail group and 5 birds from string group.

### Experience groups

We assigned juvenile jays into two experience groups: string and nail. In the string group, birds received experience of strings hanging from perches with grapes suspended at the end of one of the strings. In the nail group, birds received experience of grapes and apple slices attached to the side of a vertical wooden board, held in place by a 1 cm hidden nail. Therefore the string group received experience of visible support (the string), and the nail group received experience of hidden support (the nail). Throughout the birds’ development, string was otherwise not present in the aviary and testing compartments.

Birds received five experience sessions over 5 days at age 4 months, and over 3 days at age 5 months, and over 3 days at age 6 months. In each session, birds were given two minutes to interact with the fruit and the support. Worms were placed on the fruit to encourage interaction, as many of the birds had neophobic responses to the fruit, and in particular the string. As a result, the string group received 3 additional days experience with the string at age 6 months to facilitate habituation and to ensure they were attending to the support rather than fear of novelty. Moreover, the string group a received more worms on the fruit than the nail group because the latter group often did not require worms in order to interact with the fruit.

### Behavioural analysis of looking time

Videos were analysed using Noldus Information Technology© Observer ^®^XT software to measure total duration of looking time during each stimulus presentation. The bird was coded as looking if the top of its eye was level with the bottom of the peep hole, or higher. Because the birds had to stretch their bodies up to look, there was no point above the peep hole at which the bird could not see. If the bird rolled its head to the side (roll) or tilted it back (pitch), the bird was considered not looking if this was greater than 30 degrees. If the bird’s head was side-on to the peep hole, it was considered not looking once it turned its head (yaw) any degree further away from being parallel with the peep hole board. 81 videos (20%) were coded by a second observer, blind to the conditions. Inter-rater reliability was assessed using a two-way Intraclass Correlation Coefficient for agreement. A high degree of reliability was found between coders (ICC = 0.952, 95% confidence interval from 0.92 to 0.97 (F(80,46.8), p < 0.0001).

### Statistical analysis

For the associative learning task, the number of birds to choose the correct live object and object displayed on the monitor was analysed using exact binomial tests (two-tailed), with alpha set at 0.05.

Total looking time was analysed using Generalised Linear Mixed Models fitted to a negative binomial distribution with a log link function because data showed overdispersion. To investigate the effect of age and support on looking times for the video experiment, we ran two separate models for each position of the cheese (inside and outside) because we found a significant three-way interaction for position, support type and age. In both models, support type (contact, no contact, incorrect contact), age, presentation order and support type*age interaction were included as fixed factors.

Three separate models for the image experiment were run. For the core knowledge at age 3 months, support type (support, insufficient support, no support and incorrect support) and presentation were included as fixed terms. For each experience group (string and nails), models were run with support type (support string up (SU), insufficient support SU, insufficient support string down (SD), no support SU, no support SD, incorrect support SU, incorrect support SD) and presentation order included as fixed terms. Subject was included as a random factor for all analyses. All models were first run with all terms, and non-significant fixed terms were successively dropped from the model (least significant first) until a minimal model remained. The minimal model was one in which all the remaining terms explained the variance at p < 0.05. Minimal models were checked that they met model assumptions (homogeneity, and normality of residuals). Analyses were run in the glmmADMB^50^) and lme4^51^ packages for R Statistical Software^52^.

## Additional Information

**How to cite this article**: Davidson, G. *et al*. The development of support intuitions and object causality in juvenile Eurasian jays (*Garrulus glandarius*). *Sci. Rep.* **7**, 40062; doi: 10.1038/srep40062 (2017).

**Publisher's note:** Springer Nature remains neutral with regard to jurisdictional claims in published maps and institutional affiliations.

## Supplementary Material

Supplementary Video 1

Supplementary Video 2

Supplementary Information

## Figures and Tables

**Figure 1:**
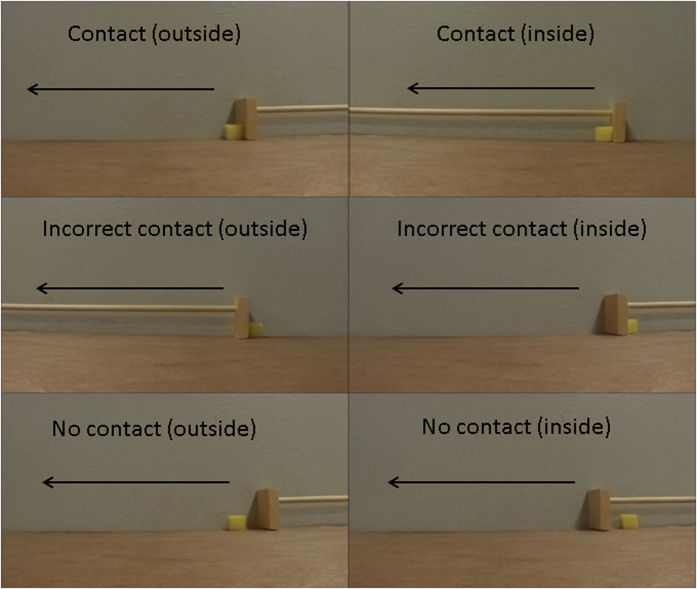
Still frames of the video stimuli. The arrow indicates direction of movement from the starting position. Birds received six video stimuli: contact (the cheese was in contact and in front of the direction of the moving rake, incorrect contact (in contact and behind the direction of the moving rake, and no contact (not in contact with the rake, but moving alongside the rake. For each of the three categories of support, the cheese was either positioned outside or inside of the rake. Therefore the position of the cheese relative to the rake differed for each contact category. Birds were first presented with a familiarisation video before the test stimuli to show the rake moving independently from the cheese (see methods and supplementary materials for videos).

**Figure 2:**
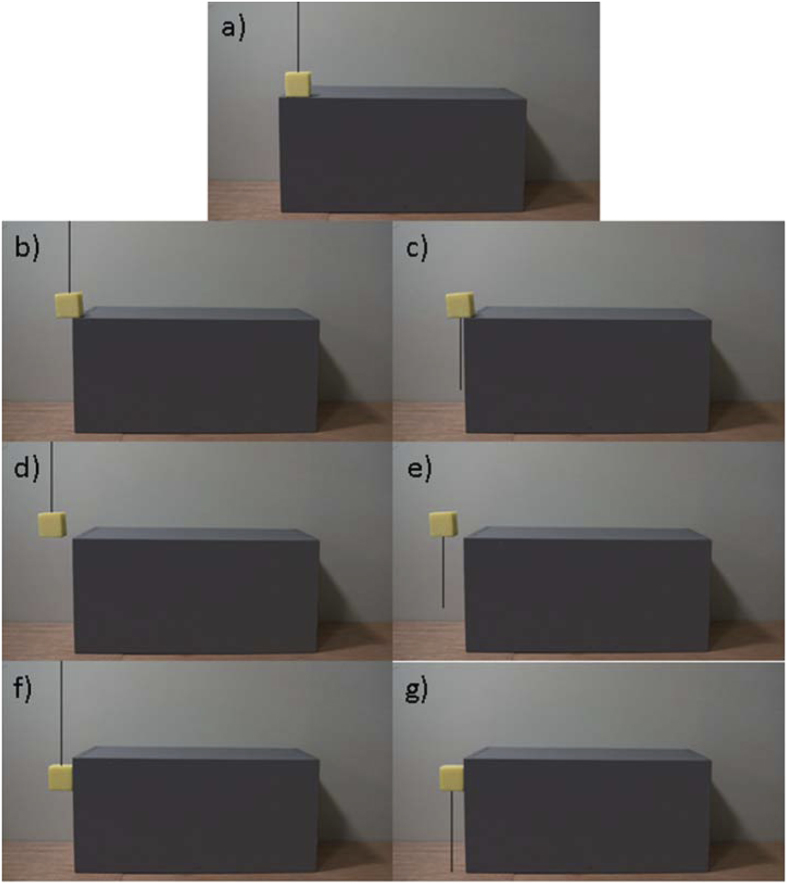
Static images of support presented to birds at age 6 months. (**a**) correct support (supported in the centre of the box), (**b,c**) insufficient support (a greater surface area of the cheese was off the box than on the box), (**d,e**) no support (the cheese had no contact with the box and was suspended in mid-air) and (**f,g**) incorrect support (the surface area of one side of the cheese was in complete contact with the vertical edge of the box). Each set of support categories is duplicated with either a string up (could be supported by string, **a,b,d,f**) or a string down (not supported by string, **c,e,g**). Birds at age 3 months were presented with four images of the same four categories of support, but without any string (see methods).

**Table 1:** Sequence of stimulus presentation across development.

Age	3 months	4 months	6 months	9 months
**Stimuli**	videos; static images (Core knowledge)	videos	videos; static images (with string)	videos
