# Erratum: The development of support intuitions and object causality in juvenile Eurasian jays (*Garrulus glandarius*)

**DOI:** 10.1038/srep42936

**Published:** 2017-02-24

**Authors:** Gabrielle Davidson, Rachael Miller, Elsa Loissel, Lucy G. Cheke, Nicola S. Clayton

Scientific Reports 7: Article number: 4006210.1038/srep40062; published online: 01 05 2017; updated: 02 24 2017

This Article contains an error in the Methods section under the subheading ‘Subjects’:

“Eurasian jays participated in the study (though see results for samples sizes for each test condition)”.

should read:

“16 Eurasian jays participated in the study (though see results for samples sizes for each test condition)”.

