# Supplementary material for: The development of support intuitions and object causality in juvenile Eurasian jays (Garrulus glandarius)
Source: Sci Rep. 2017 Jan 5;7:40062. doi: 10.1038/srep40062 (PMC5215467; doi:10.1038/srep40062)
Supplement: Supplementary Information [file srep40062-s3.pdf]

The development of support intuitions and object causality in juvenile Eurasian jays (*Garrulus glandarius*)

Gabrielle Davidson*[,a,b] , Rachael Miller[a], Elsa Loissel[a], Lucy G. Cheke[a], Nicola S. Clayton[a]

Supplementary Information

Associative learning task

Birds were presented with two objects located behind two small Perspex windows covered by a large curtain and could make a choice by flying to one of the two perches located below each window. The experimenter was out of sight behind the curtain where they moved the location of the objects (randomised across trials), raised the curtain and rewarded the birds with wax worms (rewarded object counterbalanced across birds). The experimenter monitored the birds from behind the curtain via a live video feed using a GoPro® Hero4 Black. The task consisted of three training phases (baited, multiple choices and first choice) and one test phase (live and monitor).

Training phase 1 (baited): Birds were trained to wait at a perch 1 metre away from the windows when the curtains were closed. The experimenter raised the curtain and held a worm in front of the rewarded object and the birds could fly to the perch and obtain their reward. The curtain was closed and this was repeated 10 times over 3 sessions. A maximum of two sessions were given per day, with a minimum two hour interval between.

Training phase 2 (multiple choices): This phase followed a similar procedure as the baited phase; however, birds were only presented with the reward once they landed on the "correct" perch, regardless of any mistakes. Once the bird landed on the correct perch, the experimenter presented a worm and closed the curtain. Birds received 10 trials over 4 sessions.

Training phase 3 (single choice): The curtain was raised and birds were given one choice. If they landed on the correct perch, they received a worm. If they landed on the incorrect perch the curtain was lowered without reward. Birds received 10 trails per session. In order to reach criterion and move to the test phase, birds had to choose correctly 8/10 trials over two consecutive sessions.

Test phase (live and monitor): We tested whether birds would choose the correct perch when presented with objects displayed on the monitor. The set up was the same as in training phase 3, with a monitor positioned directly behind the live objects. Birds were given four choices with the live objects (monitor as a black screen). Birds were rewarded for each correct choice to prevent extinction of the learned association. If birds did not choose all four live objects correctly, the test phase was repeated the following session. If birds chose correctly all four times, the live objects were removed and the birds were presented with one single choice of the objects displayed on the monitor.

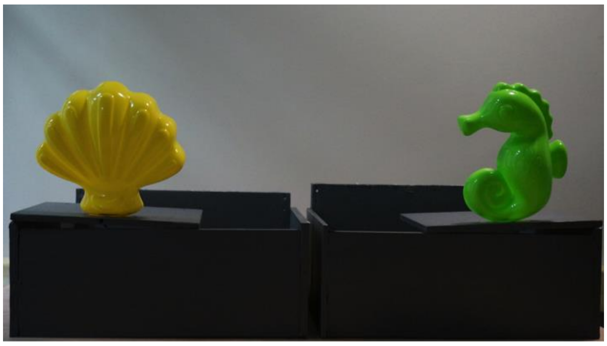

Supplemental Figure S1. Two objects (yellow clam shell and green sea horse) were revealed to the birds through two separate windows. Images of the same objects were displayed on a monitor in front of the same two windows during the test phase.

Supplemental Tables S1-S3. Bird participation for each developmental stage and for each stimulus.

Stimulus type	age (months)	Total stimuli	Total subjects	All stimuli	less 1	less 2	less 3	less 4	less 5
**Videos**	3	82	15	10	3	1	1	x	x
**Videos**	4	57	13	7	x	1	2	2	1
**Videos**	6	70	14	7	3	2	1	1	x
**Videos**	9	45	10	3	2	1	3	2	x
**Images**	3	59	16	13	2	x	1	x	x
**Images (string group)**	6	47	7	4	3	x	x	x	x
**Images (nail group)**	6	51	8	4	3	1	x	x	x

Supplemental Table S1: Total number of test stimuli (images or videos) and number of subjects at each age group. Number of subjects to have viewed all stimuli (images or videos), or less than all stimuli (minus 1,2, 3, 4, or 5 from total stimuli).

Supplemental Table S2: Number of subject participation per video stimulus and developmental stage.

Age (months)	contact push	contact pull	incorrect contact push	incorrect contact pull	no contact push	no contact pull	Mean participation
3	14	14	14	14	11	15	13.7
4	8	9	11	9	11	9	9.5
6	10	13	11	11	12	13	11.7
9	7	7	8	9	7	7	7.5

Supplemental Table S3: Number of subject participation per image stimulus and developmental stage.

Stimulus type	Age (months)	support	insufficient support	no support	incorrect support				Mean
**images**	3	15	15	14	15				14.75
		**support**	**insufficient support SU**	**insufficient support SD**	**no support SU**	**no support SD**	**incorrect support SU**	**incorrect support SD**	
**images (string group)**	6	7	7	7	7	7	7	5	6.6
**images (nail group)**	6	8	8	6	6	8	7	8	7.1

Supplemental Tables S4-S5 Results tables for GLMMs

Model	subjects	total observations	Fixed effects	β±SE	z	P
**Cheese outside**	15	124	intercept	7.28±0.19	37.72	<0.0001
**minimal model**			presentation order	-0.14±0.03	-4.69	<0.0001
			age 9 months	-1±0.25	-3.93	<0.0001
			no contact*9 months	1.03±0.36	2.84	0.005
			no contact	-0.52±0.21	-2.44	0.015

			incorrect*4 months	-0.58±0.33	-1.75	0.080
			incorrect contact	0±0.2	-0.02	0.986
			age 4 months	-0.06±0.23	-0.24	0.814
			age 6 months	-0.22±0.22	-1.00	0.316
			no contact*4 months	0.38±0.33	1.15	0.249
			incorrect*6 months	0.25±0.31	0.82	0.414
			no contact*6 months	0.36±0.32	1.14	0.253
			incorrect*9 months	0.18±0.35	0.52	0.600
**Cheese inside**	15	130	intercept	7.16±0.14	52.37	<0.0001
**minimal model**			age 4 months	-0.33±0.13	-2.57	0.010
			age 6 months	-0.23±0.12	-1.94	0.052
			age 9 months	-0.93±0.13	-6.44	<0.0001
			presentation order	-0.1±0.03	-3.71	0.0002
**dropped terms**			incorrect contact	0.04±0.11	0.35	0.728
			no contact	-0.02±0.11	-0.22	0.829
			incorrect*4 months	0.11±0.3	0.38	0.707
			no contact*4 months	-0.48±0.31	-1.55	0.121
			incorrect*6 months	0.14±0.28	0.50	0.617
			no contact*6 months	-0.16±0.28	-0.59	0.555
			incorrect*9 months	0.07±0.33	0.21	0.833
			no contact*9 months	-0.53±0.33	-1.59	0.113

Supplemental Table S4 GLMM output for looking time of video stimuli. Contact and age 3 months were set as reference categories.

Model	Subjects	Total observations	Fixed effects	β±SE	z	P
**Core knowledge (3 months)**	16	59	intercept	7.56±0.16	37.38	<0.001
**Minimal Model**			presentation order	-0.29±0.06	-4.88	<0.001
**dropped terms**			insufficient support	0.12±0.2	0.61	0.54
			no support	-0.11±0.2	-0.57	0.57
			incorrect support	-0.29±0.06 ±	0.22	0.83
**Nail group (6 months)**	15	98	intercept	6.7±0.21	32.01	<0.001
			no support string down	0.53±0.26	2.03	0.043
**Minimal Model**			insufficient string down	0.56±0.28	2.00	0.045
			incorrect string down	0.35±0.26	1.37	0.169
			incorrect string up	-0.16±0.27	-0.59	0.558

			insufficient string up	-0.1±0.26	-0.39	0.696
			no support string up	-0.05±0.28	-0.18	0.855
**dropped terms**			presentation order	-0.05±0.03	-1.46	0.145
**String group (6 months)**	15	98	intercept	6.96±0.21	33.52	<0.001
**Minimal Model**			no support string up	0.56±0.28	1.99	0.046
			insufficient string up	0.53±0.28	1.89	0.058
			incorrect string down	-0.38±0.31	-1.19	0.232
			incorrect string up	0.34±0.28	1.21	0.227
			insufficient string down	0.07±0.28	0.24	0.813
			no support string down	-0.04±0.28	-0.16	0.876
**dropped terms**			presentation order	-0.01±0.04	-0.31	0.754

Supplemental Table S5 GLMM output for looking time of image stimuli. Contact (control) was set as the reference category.

Video Legends

Video 1. Familiarisation video and six test videos: familiarisation video, contact push, contact pull, incorrect contact push, incorrect contact pull, no contact push, no contact pull. Rake direction (right to left or left to right) was consistent within individuals and counterbalanced across subjects. Video is edited from original format displayed to birds.

Video 2. Bird looking behaviour. A bird looks through the peep hole to the stimulus on the other side.
